# Impact of coupled input data source-resolution and aggregation on contributions of high-yielding traits to simulated wheat yield

**DOI:** 10.1038/s41598-024-74309-4

**Published:** 2024-10-05

**Authors:** Ehsan Eyshi Rezaei, Babacar Faye, Frank Ewert, Senthold Asseng, Pierre Martre, Heidi Webber

**Affiliations:** 1 Leibniz Centre for Agricultural Landscape Research (ZALF), Müncheberg, Germany; 2 University of Sine Saloum EL-HÂDJ IBRAHIMA NIASS, Kaolack, Senegal; 3 Crop Science Group, University of Bonn, Institute of Crop Science and Resource Conservation (INRES), Bonn, Germany; 4 Department of Life Science Engineering, Technical University of Munich, Digital Agriculture, HEF World Agricultural Systems Center, Freising, Germany; 5 LEPSE, Université Montpellier, INRAE, Institut Agro Montpellier, Montpellier, France; 6 Institute of Environmental Sciences, Brandenburg University of Technology, Cottbus, Germany

**Keywords:** Plant sciences, Ecological modelling

## Abstract

High-yielding traits can potentially improve yield performance under climate change. However, data for these traits are limited to specific field sites. Despite this limitation, field-scale calibrated crop models for high-yielding traits are being applied over large scales using gridded weather and soil datasets. This study investigates the implications of this practice. The SIMPLACE modeling platform was applied using field, 1 km, 25 km, and 50 km input data resolution and sources, with 1881 combinations of three traits [radiation use efficiency (RUE), light extinction coefficient (K), and fruiting efficiency (FE)] for the period 2001–2010 across Germany. Simulations at the grid level were aggregated to the administrative units, enabling the quantification of the aggregation effect. The simulated yield increased by between 1.4 and 3.1 t ha^− 1^ with a maximum RUE trait value, compared to a control cultivar. No significant yield improvement (< 0.4 t ha^− 1^) was observed with increases in K and FE alone. Utilizing field-scale input data showed the greatest yield improvement per unit increment in RUE. Resolution of water related inputs (soil characteristics and precipitation) had a notably higher impact on simulated yield than of temperature. However, it did not alter the effects of high-yielding traits on yield. Simulated yields were only slightly affected by data aggregation for the different trait combinations. Warm-dry conditions diminished the benefits of high-yielding traits, suggesting that benefits from high-yielding traits depend on environments. The current findings emphasize the critical role of input data resolution and source in quantifying a large-scale impact of high-yielding traits.

**Supplementary Information:**

The online version contains supplementary material available at 10.1038/s41598-024-74309-4.

## Introduction

Global food demand is estimated to increase by 62% by 2050 to meet the changing consumption needs of an expanding and more affluent population^1^. Achieving such a significant increase in food demand is not only challenging but also threatened by climate change^2,3^. The rising frequency and intensity of droughts, heatwaves, and floods observed worldwide in recent years have negatively impacted crop yields and are projected to persist^4,5^. Global wheat yield is projected to increase between 9% and 18%, depending on the greenhouse gas emission scenarios^6^. However, wheat yield has stagnated or even declined in recent decades across various regions^7^. A 1.88% per year yield gain is required to meet the projected demand to increase wheat production^8^ which is currently varying between 0.5% and 1%^9^. Therefore, the development of climate-resilient and high-yielding cereal cultivars is essential to support future food security^10^.

Higher radiation use efficiency (RUE) and fruiting efficiency, homogenous light distribution across the canopy (using light extinction coefficient), and larger grain weight potential were defined as crucial traits that could improve wheat yield^11^. The majority of long-term wheat yield improvements are linked to optimizing the harvest index for greater carbon partitioning to yield without significantly altering total biomass accumulation^12^. Harvest index improvements might have reached its limits^13^ as recent efforts to increase yield have primarily focused on increasing total biomass growth^14^. The potential of RUE for improving wheat biomass has not been fully utilized yet^15^. Improvement of RUE (as a complex trait) could be achieved by different processes such as a change in canopy photosynthesis efficiency, and enhanced carbon translocation^16^. Source limitations have been reported for modern wheat cultivars with a high harvest index^17^. However, higher biomass production by improved RUE would increase the relevance of the sink strength in keeping the source-sink balance^18^. An increment in biomass production should thus be accompanied by higher fruiting efficiency (FE) defined as grains set per unit of spike dry weight^19^ to achieve a grain yield increase^20^. The light extinction coefficient (*k*) determines the efficiency of light interception within the canopy profile^21^. More erect leaves lead to greater light penetration into the canopy profile increasing canopy RUE^22^. Disentangling the complex relationships between those traits (RUE, FE, and *k*) and the environment is critical for guiding the breeding efforts in developing climate-resilient/high-yielding cultivars^23^. However, improving our mechanistic understanding by empirical experimentation only, is challenging due to costs, risks, and scaling issues.

Process-based crop models are robust tools to interpret genotype × environment interactions from the results of experimental platforms^24,25^. The majority of these models are typically developed at the field scale, and upscaling their outcomes to regional or global scales amplifies uncertainty^26^. High-resolution model inputs are usually not available at larger scales, therefore, aggregated weather, soil, and crop data are often used for such simulation experiments^27^. Or outputs of crop models at high resolution are aggregated to a district, region, or a country, providing an overview for larger scales^28^, same as agricultural statistics obtained primarily from field scale and aggregate to a region or country. A few studies showed significant effects of weather data aggregation on crop model results, in spatially heterogeneous environments^29^. Most studies highlighted soil data as the primary source of aggregation error^30–32^. Weather input data source is another basis of uncertainty for large-scale modeling experiments^33^. Uncertainty of model results due to data source have been captured for both weather^33^ and soil^34^ inputs. However, the effects of data sources on crop model results have been less intensively studied than data aggregation impacts.

Crop models have already been implemented in genotype to phenotype pipelines at the field scale to explore the contribution of those traits to crop yield at larger spatial scales^35^. However, little is known about how the combined effect of input data sources and their associated resolutions (hereafter referred to as “resolution-sources”), along with data aggregation, influences the contribution of high-yielding traits to simulated yield. To narrow the knowledge gap, the presented research aims (i) to measure the effects of using different input data sources with different resolution-sources (field, 1 km × 1 km, 25 km × 25 km, and 50 km × 50 km) on simulated yield with variations of radiation use efficiency, fruiting efficiency, and light extinction coefficient for winter wheat, and (ii) to investigate the influence of output data aggregation on simulated yield with diverse trait combinations.

## Methods

### General workflow of study

**Fig. 1:**
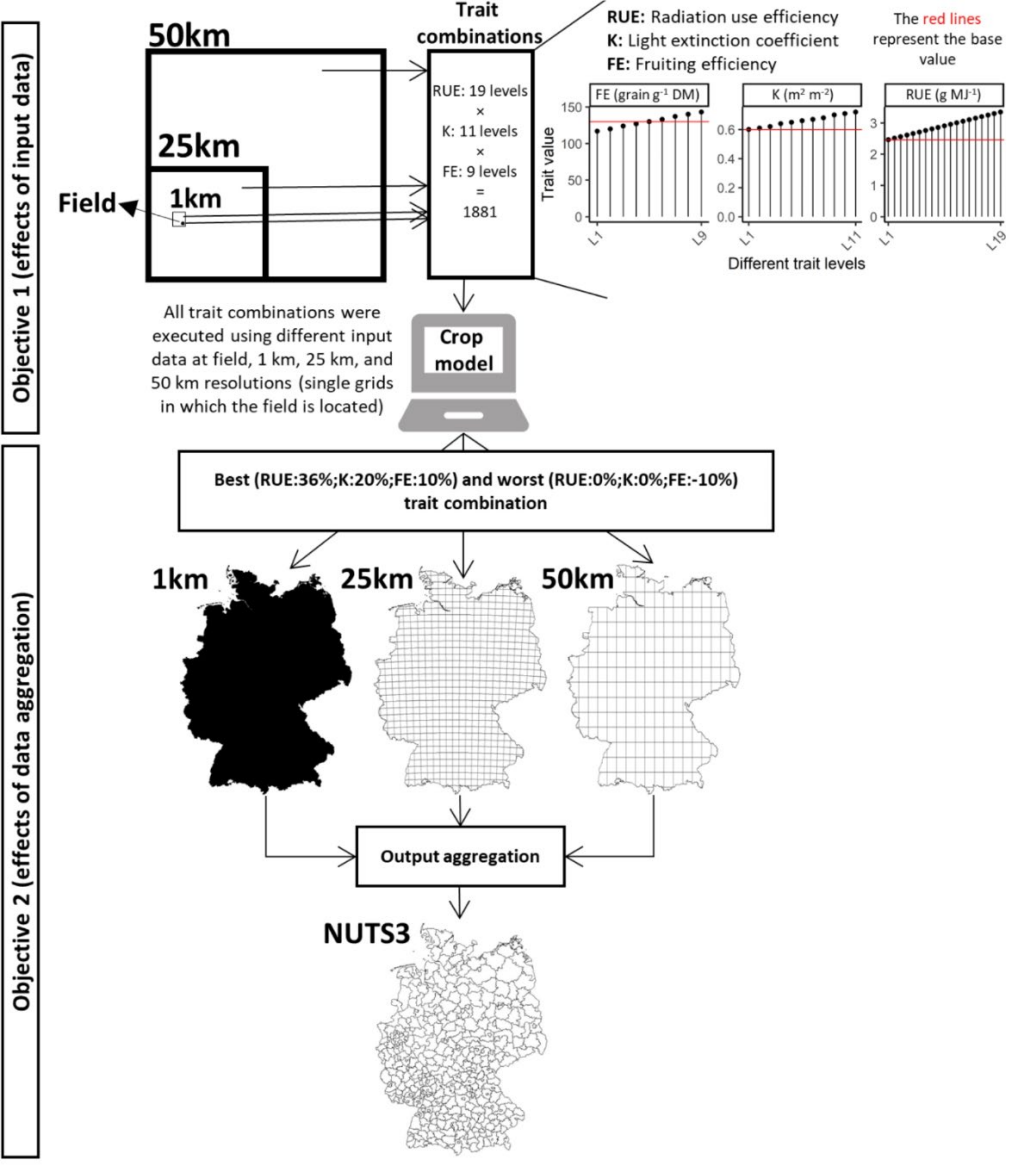
Schematic overview of the progressing steps to address the study’s objectives. The steps for objective 1 involved simulating 1,881 trait combinations with the crop model, using various field-scale data and grid resolutions of 1 km x 1 km, 25 km x 25 km, and 50 km x 50 km. For objective 2, the steps included running the crop model with both the best and worst trait combinations for all grid resolutions throughout Germany. The simulated yield was then aggregated to the NUTS3 level. The maps were generated with R package terra, version 1.7-78 using shapefiles from the federal states obtained from the Federal Agency for Cartography and Geodesy of Germany (http://www.geodatenzentrum.de).

The study workflow was designed to address both objectives (see above) using a modeling platform (SIMPLACE)^36^ and input data at different resolution-sources and trait combinations (Fig. 1). The weather and soil data were developed or extracted at 1 km, 25 km, and 50 km for Germany from different data sources. A factorial combination of traits (1,881 combinations) was developed^24^ based on the observed potential of RUE, FE, and K of high-yielding wheat double haploid (DH) lines^37^. A modeling solution was executed using diverse scenarios under rainfed conditions. First, wheat growth and yield were simulated at the field scale and the single grids in which the field was located from 1 km, 25 km, and 50 km weather/soil datasets for all combinations of traits in the period 2001–2010. Second, the modeling solution was implemented for 1 km, 25 km, and 50 km across Germany using best and worst trait combinations (based on the results of the first step). The model outputs were aggregated to the Nomenclature of Territorial Units for Statistics Level 3 (NUTS3) scale to quantify the effects of data aggregation (Fig. 1).

### Weather and soil data

The crop model environmental inputs have been described in detail in various studies including^38^. They included daily weather (minimum and maximum temperatures, radiation, precipitation, and wind speed) and soil characteristics (volumetric (%) crop available water at permanent wilting point, field capacity, saturation, bulk density, soil organic carbon and rooting depth) at different resolutions were obtained from various sources in the period 2001–2010 across Germany. Field-scale model inputs (weather and soil) were obtained from an agricultural experimental station located in Northern Germany (54.53°N, 9.55°E). The weather data at 1 km were acquired by interpolating weather station data from the German weather service (DWD)^29^. The 25 km resolution weather data were extracted from the Joint Research Center’s (JRC) Agri4Cast database (version 1.0)^39^. Median of five CMIP6 global circulation models (IPSL-CM6A-LR, UKESM1-0-LL, GFDL-ESM4, MPI-ESM1-2-HR and MRI-ESM2-0) from Inter-Sectoral Impact Model Intercomparison Project (ISIMIP) was employed as weather data inputs at 50 km resolution.

The soil data at 1 km resolution were derived from soil reconnaissance (BÜK1000N) by the Federal Institute for Geosciences and Natural Resources (BGR) based on the aggregation of the dominant soil type^38^. European Soil database from the JRC European Soil Data Portal (http://eusoils.jrc.ec.europa.eu/) was used to derive the soil data at 25 km resolution. The Corine Land Cover 2000 was used to mask out non-croplands (Webber et al., 2018^39^). The grid cells marked as non-cropland were masked out according to the Corine land cover 2006^40^ for 1 km resolution. The soil data at 50 km resolution were derived from the Harmonized World Soil Database (ESDB) which was originally 30 arc-second (≈ 1 km), aggregated at 50 km based on the procedure explained in Jägermeyr et al.^6^).

### High-yielding traits

The high-yielding wheat traits were developed from the difference in RUE, K, and FE between a check cultivar (Bacanora) and the two highest yielding lines of the Bacanora × Weebil DH population^37,41^. The improvement in RUE, K and FE traits were up to + 34%, + 10% and − 5%, respectively^24,42^. The ranges of each trait are defined based on those measurements, which resulted in 1,881 unique trait combinations including 19, 11 and 9 levels of RUE, K and FE, respectively (Fig. 1). Improved high-yielding traits were obtained under optimized nutritional (300 kg Nha^− 1^, 300 kg P_2_O_5_ha^− 1^, and 150 kg K_2_Oha^− 1^) and agronomic management (including proper irrigation and controlling pest and disease) conditions^37^. The base values for RUE, K and FE were 2.46 g dry mass MJ^− 1^ PAR, 0.6 m^− 2^ ground m^− 2^ leaves and 130 grain number g^− 1^ spike dry mass, respectively^42^.

### Crop model setup

Scientific Impact assessment and modeling platform for advanced crop and ecosystem management (SIMPLACE) is a modeling framework based on the solution concept, including discrete and interchangeable modules^36^. It has been broadly employed in process understanding and impact assessment studies, particularly in capturing heat, drought, and nitrogen signals on crop growth^30,39,43^. SIMPLACE was developed by pairing the Lintul-5 coupled with SlimWater, the FAO-56 Penman-Monteith for evapotranspiration estimation, and a canopy temperature module model as input of the heat stress module described by Webber et al.^38^. The SIMPLACE modeling platform was chosen because it performed relatively similarly to the multi-model ensembles in previous studies^44^.

In SIMPLACE, crop development is driven by daily mean air temperatures modified by photoperiod and vernalization (for winter crops)^45^. Biomass accumulation is initially driven by light interception as a function of the green leaf area, then transformed into dry matter using the radiation use efficiency concept modified by the phenological stage. Heat stress impacts grain yield when the estimated canopy temperature exceeds the defined temperature threshold (31 °C for wheat) around anthesis. Water stress is modeled by using the ratio between actual and potential transpiration affecting leaf area expansion, carbon partitioning to different organs, and RUE. The crop model was not parametrized for yield, but wheat phenology and sowing/harvest dates were calibrated using the German weather service database at grid scale^46^. Nitrogen stress was not included in these simulations, assuming in general well fertilized wheat crops. However, heat and drought stress were stressors affecting crop growth. Simulated yields are reported at 0% moisture.

### Simulation scenarios

To address the study’s first objective, the modelling solution was executed for field and single grid cells from different input datasets (1 km, 25 km, and 50 km) where the field was located in Schleswig–Holstein, Germany using all trait combinations for the period 2001–2010. The worst (RUE:0%;K:0%;FE:-10%) and best (RUE:36%;K:20%;FE:10%) trait combinations^24^ were selected based on the median yield at the first step and used for the second step of simulations at Germany scale. The country wide simulations were conducted using those two trait combinations for all study resolution-sources addressing the second objective of the study. The simulated yield for each resolution-source was aggregated (output aggregation) to the NUTS3 scale by averaging the yield for grids inside each polygon, quantifying the effects of data aggregation on simulated yield. The aggregation procedure for the 25 km and 50 km resolutions was performed using an area-weighted approach.

### Data analysis

Summary statistics, frequency distributions, and kernel probability density of simulated yields at different input resolution-sources were calculated, quantifying the importance of each trait and data aggregation effects. Spatial patterns of simulated yields were compared between 1 km and other resolutions by calculating the absolute difference (AD) and the difference (D) at aggregated NUTS3 scale. In addition, the mean of absolute differences and the mean of differences across NUTS3 units were calculated. As AD is always positive, it is possible to measure the agreement of maps at the polygon scale by the mean of AD across all polygons. The mean of D was computed across all polygons to indicate systematic bias between aggregated maps. A segmented, piecewise linear regression^47^ was used to explore the yield response to climate and soil variables. Through maximizing the combined coefficient of determination (R^2^) of the two linear regression estimates before and after the breakpoint, one single break point was detected^48^. All data analysis was conducted using RStudio (R version 4.0.4).

## Results and discussion

### Impact of high-yielding traits on simulated yield across different input data resolution-sources

**Fig. 2:**
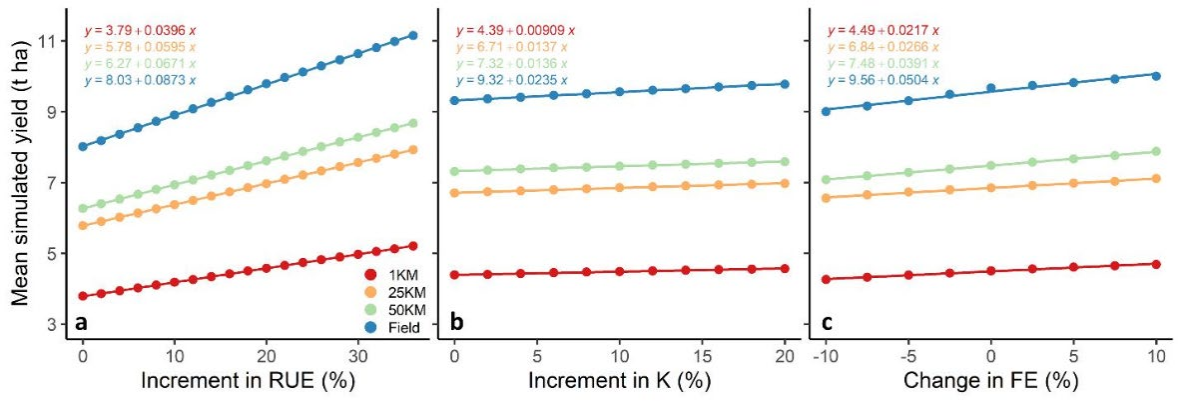
Mean simulated yield (2001–2010) for various levels (increment from base cultivar) of radiation use efficiency (RUE) (**a**), light extinction coefficient (K) (**b**), and fruiting efficiency (FE) (**c**) executed at field, 1 km, 25 km and 50 km resolution-source (single grids in which the field is located) input data.

As in the study of Stella et al. (2023)^24^ across many environments, results here for RUE showed large impacts on simulated yield for the various resolution-sources input data (Fig. 2). Increments in RUE compared to the base cultivar increased the yield by up to 2.28 t ha^− 1^ (as mean over resolutions). However, increments in K and FE resulted in remarkably less (< 0.4 t ha^− 1^) yield improvement (Fig. 2). Field scale data resulted in the strongest response to change for all high-yielding traits (0.02 to 0.09 t ha^− 1^ increase per % of trait increment), while 1 km input data resulted in the least (0.01 to 0.04 t ha^− 1^ increase per % of trait value increment) yield improvement (Fig. 2). Yield response to high-yielding traits using 25 km and 50 km inputs were between field and 1 km (Supplementary Fig. 1).

Despite the large differences in simulated yield resulting from various input data resolution-sources, the ranking of high-yielding traits was not affected. Superior traits (and their sizes) consistently led to higher yields. Although, the impact of RUE increment as the most influential trait on yield (0.09 t ha^− 1^ increase per % of RUE increment) notably receded when the input data resolution-source shifted from field scale to 1 km scale (0.04 t ha^− 1^ increase per % of RUE increment). The differences in performance of high-yielding traits were also confirmed by testing their potential across diverse environments^24^. Some literature demonstrated the significant potential of increased RUE to enhance yield by promoting biomass growth^14,49^. The improvement in grain yield of the best yielding DH lines was 54% higher than that of the check cultivars (Invento-BAER), mainly explained by increase in grain number and RUE^37^. The results of the same experiments on high-yielding DH lines showed a curvilinear relationship between grain yield and grain number, which could lead to a decline in the potential of grain number for future yield improvement^37^. It is also possible that having more erect flag leaves may not improve overall light interception. The ideal canopy structure is conceptualized by a moderate leaf angle at the bottom and erect leaves at the top of the canopy^50^.

**Fig. 3:**
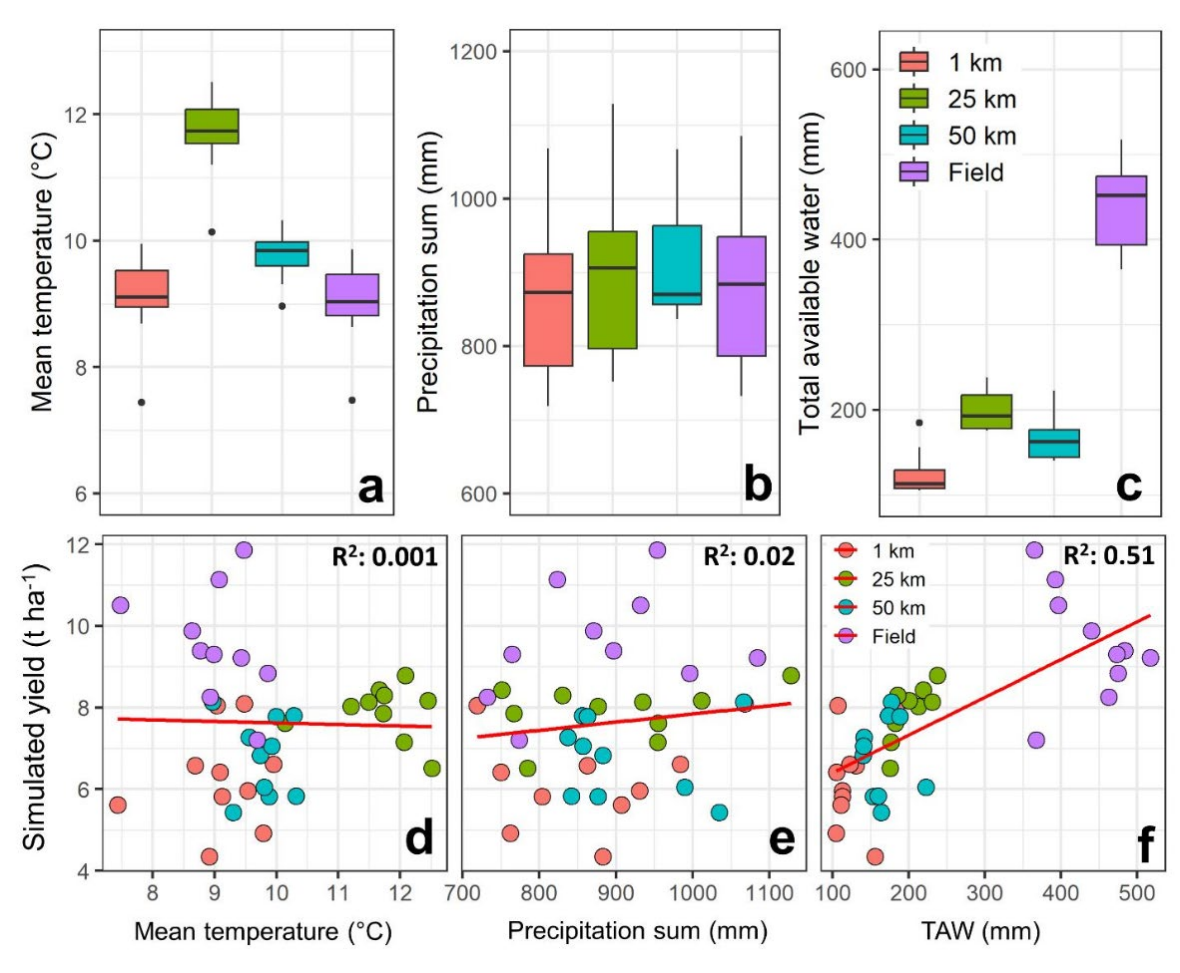
Boxplot comparing the mean temperature, precipitation sum during growth period, and total available water (TAW) for model inputs at field, 1 km, 25 km, and 50 km resolutions for the gird grid cell corresponding to the field plot considered in this study for the 2001–2010 period (**a**–**c**). (**d**–**f**) Relationships between yield and model inputs across various model resolutions and sources as input. Total available water is simulated in the SlimWater^54^ component of the model, which considers the volumes of crop water uptake, soil evaporation, surface runoff, and percolation below the root zone.

There was a significant variation in the simulated yield, with the highest yield resulting from field inputs and the lowest yield derived from 1 km resolution input data, regardless of the range of traits (Fig. 2). Comparison of the precipitation sum from various input resolutions and sources revealed no substantial difference, ranging from 867 mm per year to 910 mm per year (Fig. 3). However, there was a remarkable variation in mean temperature (from 9.1 °C to 11.7 °C) and total available soil water (125 mm to 437 mm) during the growth period among different input resolutions and sources (Fig. 3). Analysis of the relationships between model inputs and simulated yields revealed that total available water (TAW) was the main factor explaining yield variation (R^2^ = 0.51), considerably more than mean temperature (R^2^ = 0.001) and precipitation sum (R^2^ = 0.02; Fig. 3). Exploring the correlation between the difference in simulated yield (field minus other resolution-sources) and the difference in various input data (input from the field minus inputs from other resolution-sources) indicated that the difference in TAW showed the highest correlation with yield difference at 1 km (-0.61) and 25 km (-0.58) resolutions (Supplementary Fig. 2). However, the difference in mean temperature shows the highest correlation with yield difference for the 50 km (-0.55) resolution (Supplementary Fig. 2). The differences in TAW among resolutions, being higher with field data used as model input and much lower with 1 km data as input, may explain the large differences in simulated yields among resolutions shown in Fig. 2. This indicates the importance of soil data as the main driver of yield differences when altering input resolution and sources. Other scaling studies have also highlighted the significance of soil data on simulated yield compared to climate data^30,51^. Our analysis (Supplementary Fig. 2) reveals that yield-driving factors can be scale-specific. The correlation between yield differences (field vs. 50 km resolution) and TAW differences was notably lower (-0.27) compared to mean temperature differences (-0.55). This indicates that primary yield-driving factors can switch depending on the simulation scale and the response function to the input variable in the model. Such switches in yield-driving factors have been previously observed in different nutrition treatments^52^, supporting our findings. However, the response of simulated yield to changes in traits on was not considerably influenced by weather or soil data resolution and source. However, soil data uncertainty can potentially overshadow the response to climate on projected yields in impact assessment studies^53^.

**Fig. 4:**
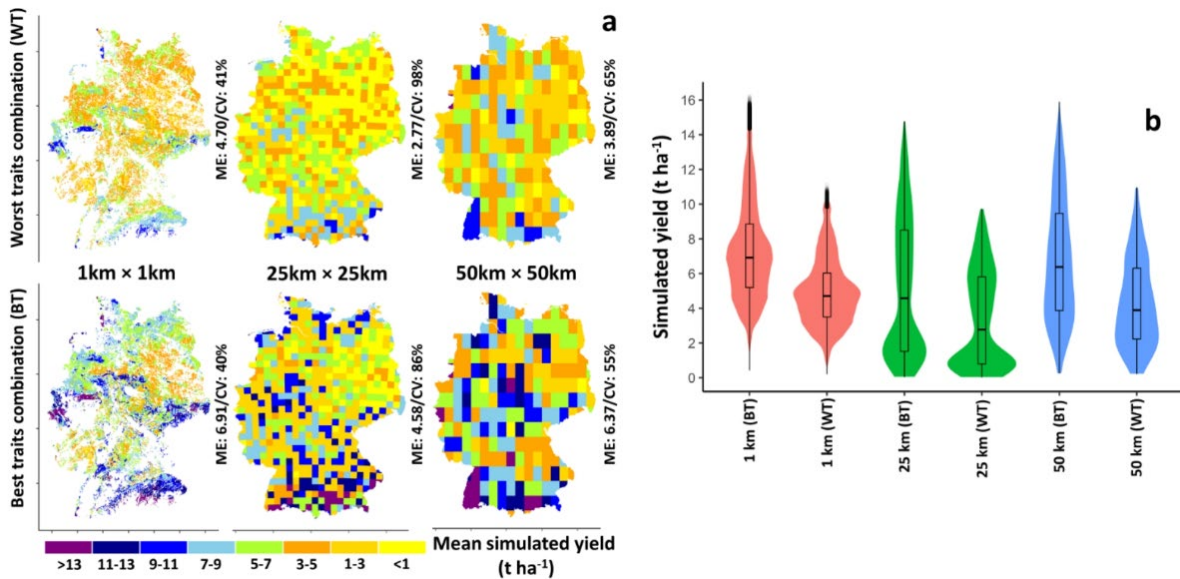
The grid scale spatial pattern (**a**) and violin/box plot (**b**) of simulated yield (2001–2010) for worst (WT; RUE: 0%; K: 0%; FE: − 10%) and best (BT; RUE: 36%; K: 20%; FE: 10%) traits combinations using 1, 25, and 50 km resolution-source input data. *ME* mean, *CV* coefficient of variation. The maps were generated with R package terra, version 1.7-78 using shapefiles from the federal states obtained from the Federal Agency for Cartography and Geodesy of Germany (http://www.geodatenzentrum.de).

The results of large-scale simulations across Germany showed a large influence of input data resolution and sources on the simulated yield for the worst and best trait combinations (Fig. 4). Using 1 km resolution as input data resulted in the highest country-wide mean simulated yield (4.70 t ha^− 1^ and 6.91 t ha^− 1^) with little difference in spatial variability (40% and 41%) for both trait combinations (Fig. 4a). On the other hand, implementing the crop model with 25 km resolution input data led to the lowest simulated mean yield (2.77 t ha^− 1^ and 4.58 t ha^− 1^) and highest spatial yield variability (86% and 98%) (Fig. 4a). The variability of simulated yields (indicated by interquartile range) was higher for the best trait combinations (3.7, 7.0, and 5.6 t ha^− 1^ for 1, 25 and 50 km, respectively) than for the worst trait combinations (2.5, 5.0, and 4.1 t ha^− 1^, for 1 km, 25, and 50 km, respectively) for all input data resolution-sources (Fig. 4b). Comparing the spatial patterns of simulated yield for both the best and worst trait combinations with the spatial pattern of observed yield (reported yield statistics^55^) revealed that the model accurately captured the lower yields in eastern Germany and higher yields in the western parts. However, it overestimated the yield in the southern parts of the country (Supplementary Fig. 3). The model generally failed to account for the negative impacts of excessive rainfall events^38^, which are more prevalent in the southern parts of the country. The relatively low accuracy of the model in capturing the observed yield (Supplementary Fig. 3) could be attributed to the fact that the model was only calibrated for phenology. This calibration approach was chosen because the main objective of the study was to capture the yield response to changes in high-yielding traits, rather than to produce the best estimation of observed yield. Also, variability in management such as sowing data and in maturity type of varieties not considered in the simulations may have contributed to the deviation of model simulations from observations. Comparing the distribution of mean simulated yield for each NUTS3 unit using different source-resolutions and best and worst trait combinations across Germany with observed yield indicated a significant difference between simulated and observed yield distributions for all simulation options. However, the worst trait combination in 1 km and 50 km source-resolutions showed better overlap with the observed distribution (Supplementary Fig. 4). This suggests that the model overestimated yields when using the best trait combination, while the worst trait combination might better represent the average conditions across diverse NUTS3 units.

**Fig. 5:**
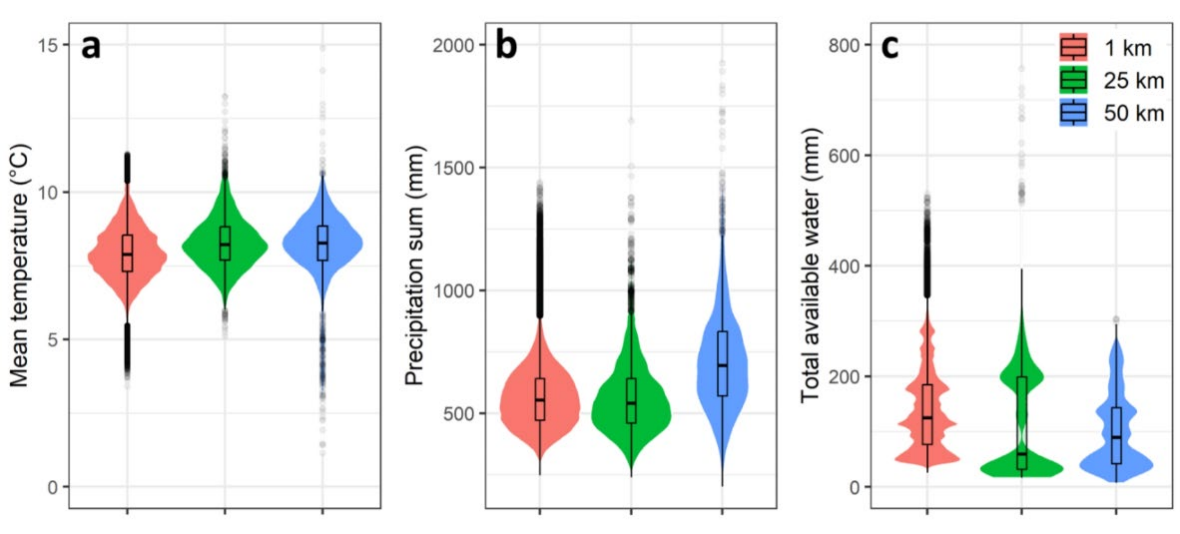
Violin/box plot of the mean temperature (**a**), annual precipitation sum (**b**), and total available water (**c**) during the growth period (emergence to maturity) for 1, 25, and 50 km input data resolution-source for all soil-weather grids nationwide.

**Fig. 6:**
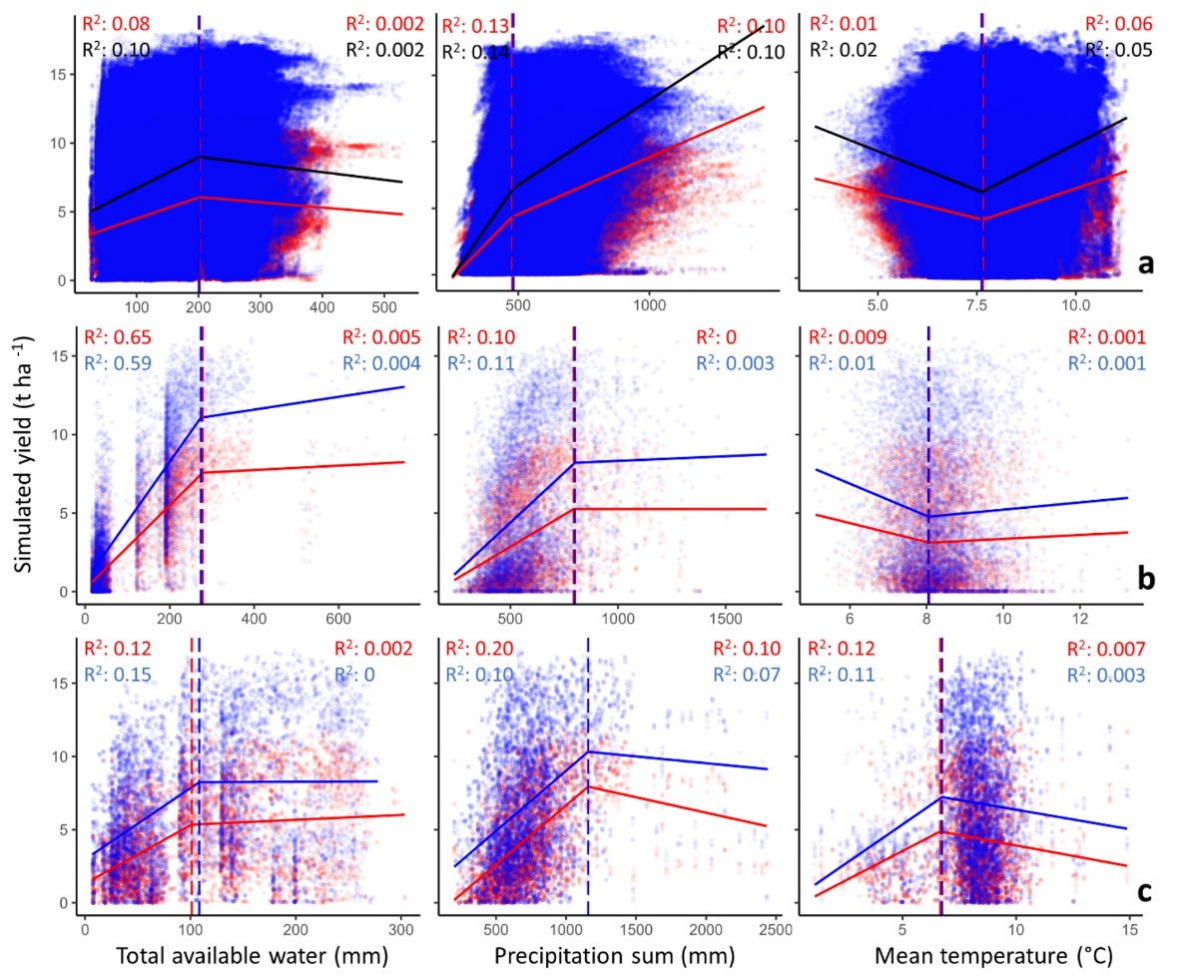
Segmented piecewise regression between crop yields simulated at 1 km (**a**), 25 km (**b**), and 50 km (**c**) source-resolution and total available water, seasonal precipitation sum, and mean temperature during the growing season for the worst (RUE: 0%; K: 0%; FE: − 10%—presented in red) and best (RUE: 36%; K: 20%; FE: 10%—presented in blue) trait combinations in the period 2001–2010. The red and blue lines show the estimated break points for the segmented regression. The R^2^ values represent the coefficient of determination before and after the estimated break point.

Seasonal mean temperatures at different spatial resolutions showed substantial overlap in their distribution with differences in medians (Fig. 5a). The median seasonal precipitation sum was 20% higher at a 50 km resolution than at other data resolutions (Fig. 5b). The cumulative available soil water at a 1 km resolution was 53% and 29% higher than those at 25 and 50 km, respectively (Fig. 5c). While the spatial patterns of those variables appear relatively similar, the value ranges differ notably (Supplementary Fig. 5). The processing of model outputs indicated that the responsiveness of simulated yield to weather and soil variables is related to the source-resolution of input data (Fig. 6). Precipitation sum during the growth period showed the largest impact on simulated yield variability (R² = 0.10 and 0.14) when using 1 km data as model input (Fig. 6a). However, simulated yield at 25 km was more affected by soil total available water (R² = 0.65 and 0.59) than by the variability of precipitation sum and mean temperature during the growth period for both the worst and best trait combinations (Fig. 6b). Soil water and precipitation sum indicated relatively similar impacts on simulated yield when the model was executed using 50 km inputs (Fig. 6c). The trait combinations did not remarkably influence the yield response to the soil and weather variables in all resolutions, and the estimated breakpoints were relatively similar between best and worst trait combinations (Fig. 6).

Experimental evidence has shown the adverse potential of terminal drought on significant suppression of the high-yielding traits of wheat without a remarkable impact of high temperature^56^. The yield advantage of high-yielding wheat genotypes (with higher biomass production as a driver) under optimal conditions was largely suppressed by drought stress^57^. Large-scale simulations have also highlighted the importance of water (soil characteristics and precipitation sum) as the primary drivers of input uncertainty^53^ and yield variability^58^.

### Effect of output data aggregation on simulated yield

**Fig. 7:**
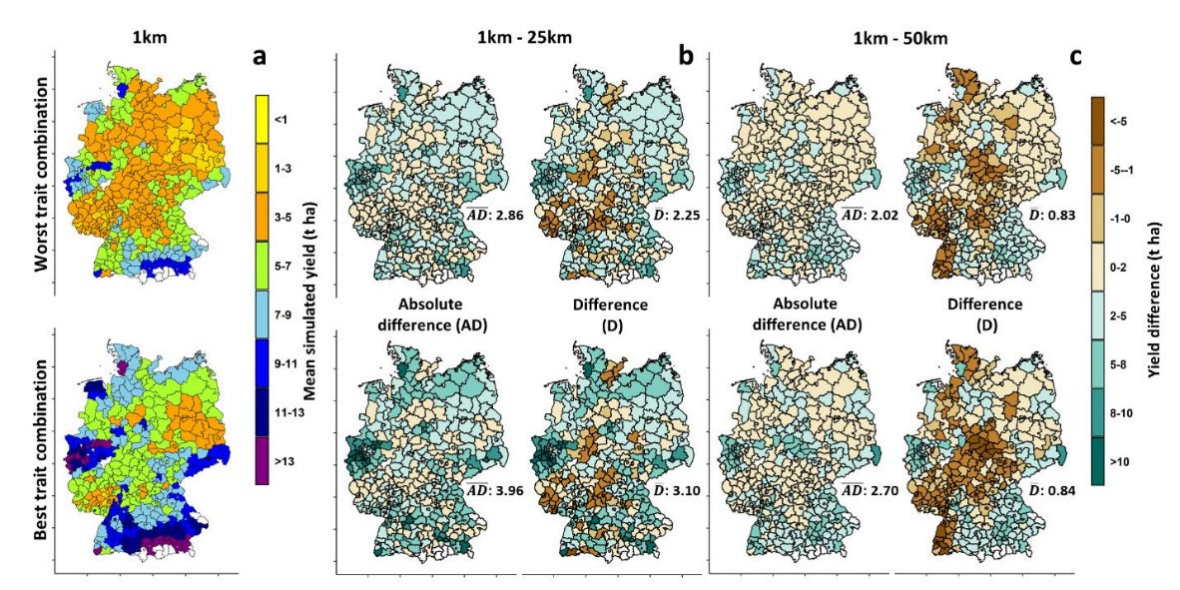
Average simulated yield aggregated from 1 km to NUTS3 scale using worst (RUE: 0%; K: 0%; FE: − 10%) and best (RUE: 36%; K: 20%; FE: 10%) trait combinations (**a**). The maps in panels (**b**, **c**) illustrate the absolute (AD) and regular (D) differences in aggregated yield (grid to NUTS3 scale) between 1 km and 25 km (**b**)/50 km (**c**) resolutions for the period 2001–2010, using both the worst and best trait combinations. The maps were generated with R package terra, version 1.7-78 using shapefiles from the federal states obtained from the Federal Agency for Cartography and Geodesy of Germany (http://www.geodatenzentrum.de).

Model input resolution-source had a slight impact (0.09 to 0.68 t^− 1^ ha) on average simulated yield (2001–2010) aggregated at NUTS3 level for both worst and best traits combination (Fig. 4 & supplementary Fig. 6). Yield variability (measured by the coefficient of variation) at NUTS3 level was comparable for the different input data resolution-sources. The mean absolute difference ($\:\stackrel{-}{AD}$) and difference $\:\stackrel{-}{(D})\:$between 1 km and other resolutions were remarkably higher for 25 km ($\:\stackrel{-}{AD}$ = 2.86–3.96 t^− 1^ ha^− 1^ and $\:\stackrel{-}{D}$ = 2.25–3.10 t^−1^ ha^−1^) compared with 50 km, particularly for the best trait combination (Fig. 7). There was no systematic spatial yield pattern change observed between the 1 km and 50 km resolution sources, as both positive and negative differences were present, and the mean difference was remarkably smaller than the mean absolute difference (Fig. 7). However, using weather and soil datasets at 25 km as model input resulted in a systematic difference compared to 1 km input data in the spatial pattern of simulated yield because $\:\stackrel{-}{AD}$ was close to $\:\stackrel{-}{D}$ (Fig. 7). The greatest spatial yield difference (1–25 km) was obtained in the western and central parts of the country (Fig. 7).

**Fig. 8:**
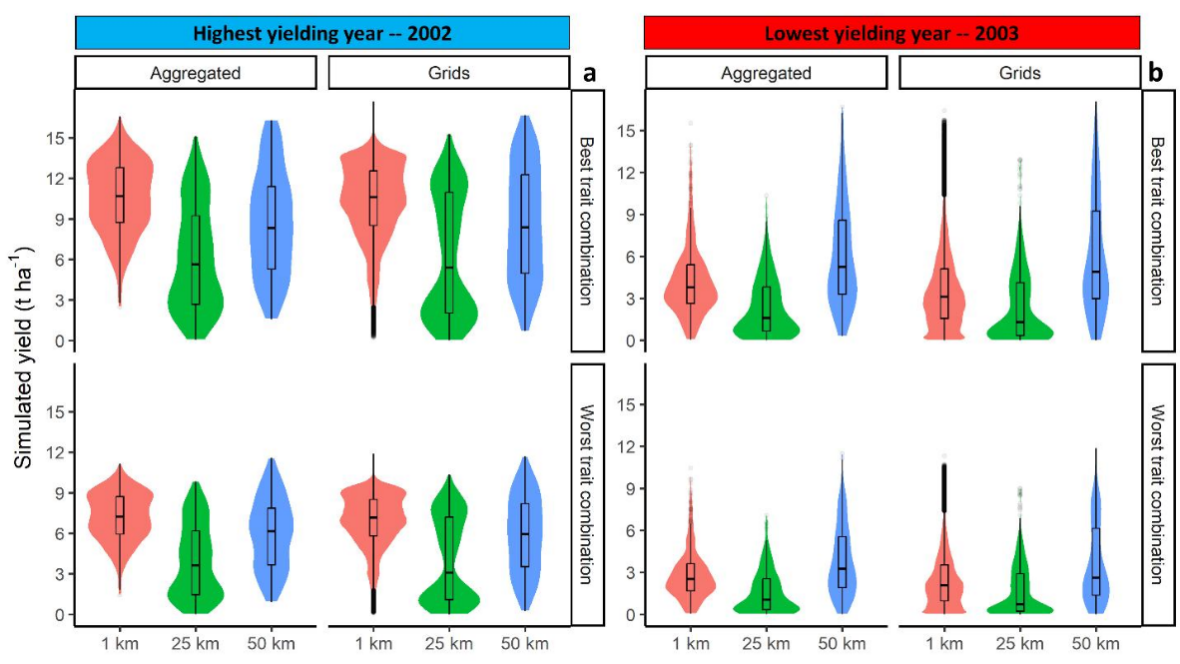
Violin/box plot of aggregated and grid level simulated yield for the highest (**a**) and the lowest (**b**) yielding year, based for the worst (RUE: 0%; K: 0%; FE: − 10%) and best (RUE: 36%; K: 20%; FE: 10%) trait combinations simulated with input data at 1, 25, and 50 km resolution-source. Extreme low and the highest yielding years were selected based on the median simulated yield at 1 km resolution.

There were large differences in simulated yield between the extreme low (2003) and best yielding year (2002) years (Fig. 8). Yield aggregated at NUTS3 level increased by 63% (4 t ha^− 1^) and 59% (5 t ha^− 1^) tags from extreme low to the best yielding year for the worst and best trait combinations, respectively (Fig. 8). The impact of high-yielding traits on simulated yield aggregated at NUTS3 level was limited to 34% (1.96 t ha^− 1^; Fig. 8). Nevertheless, data aggregation resulted in a slight increase in mean simulated yield of 0.2 t ha^− 1^ (for the best yielding year) to 0.5 t ha^− 1^ (for extreme low yield year; Fig. 8). The current results confirm the findings of previous studies on the minor effects of output data aggregation on mean simulated yield, particularly when soil data aggregation (different data sources were implemented in the current study) was not involved^28,29,59^.

## Conclusion

The resolution and sources of input data did not alter the ranking of high-yielding trait impacts on grain yield, but they did influence the potential for yield increase achieved by implementing those traits. Nonetheless, it is essential to acknowledge that the outcomes might have been substantially dissimilar for adaptive traits, such as RUE response to water deficit or change in stomatal conductance to vapor pressure deficit. The impact on yield of RUE was much more substantial than that of K or FE for all input data resolution and sources. However, the yield improvement rate per percent increase in RUE was resolution-specific. Our results suggest that water related inputs affecting plant water availability is the primary driver of simulated yield variability (compared to temperature) among the various model input datasets. However, it did not change the contribution of high-yielding traits to increasing yield, which means that yield-increasing trait combinations always results in higher yield simulations regardless of input data resolution and source. Data aggregation had a minor effect on the simulated yield for different trait combinations. Finally, warm-dry conditions (such as year 2003) can mostly suppress a positive impact of high-yielding traits regardless of input data resolution-sources. Changing the model input data resolution-source from field to 50 km affects the potential size of yield improvement from high-yielding traits, highlighting the need for high-quality input data and a tailored approach and careful interpretation when upscaling simulation results from the field to regional scales. Future research should aim to test the potential of high-yielding traits under more extreme seasonal growing conditions and under sub-optimal management, where yield benefits might be low, using various model input sources and resolutions. In addition, it should be noted that current crop models cannot fully address the interactions among RUE, the K, and FE due to the complexity of these interactions and the lack of process understanding of the dynamics of source-sink relationships^60^. This limitation suggests the need for repeating such modeling experiments using a new generation of models that better consider these interactions.

## Electronic supplementary material

Below is the link to the electronic supplementary material.

Supplementary Material 1

## Data Availability

The datasets used and/or analysed during the current study available from the corresponding author on reasonable request.
