# Supplementary material for: Impact of coupled input data source-resolution and aggregation on contributions of high-yielding traits to simulated wheat yield
Source: Sci Rep. 2024 Oct 5;14:23172. doi: 10.1038/s41598-024-74309-4 (PMC11455967; doi:10.1038/s41598-024-74309-4)
Supplement: Supplementary file 1 — Supplementary Material 1 [file 41598_2024_74309_MOESM1_ESM.pdf]

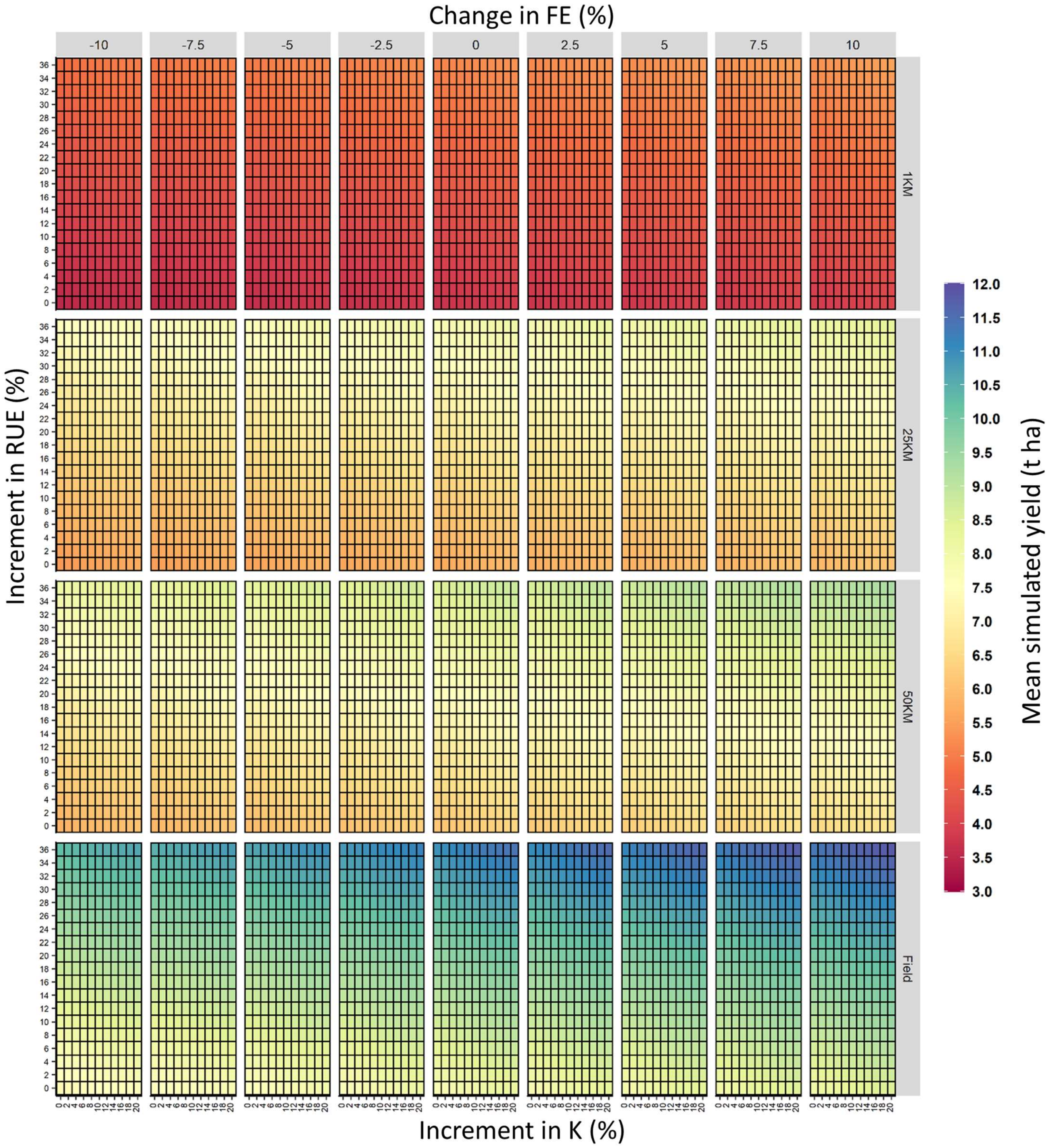

Supplementary figure 1. Mean simulated yield (2001-2010) for all trait combinations (1881) among various levels of radiation use efficiency (RUE), light extinction coefficient (K), and fruiting efficiency (FE) executed at field, 1 km, 25 km and 50 km resolution-source input data.

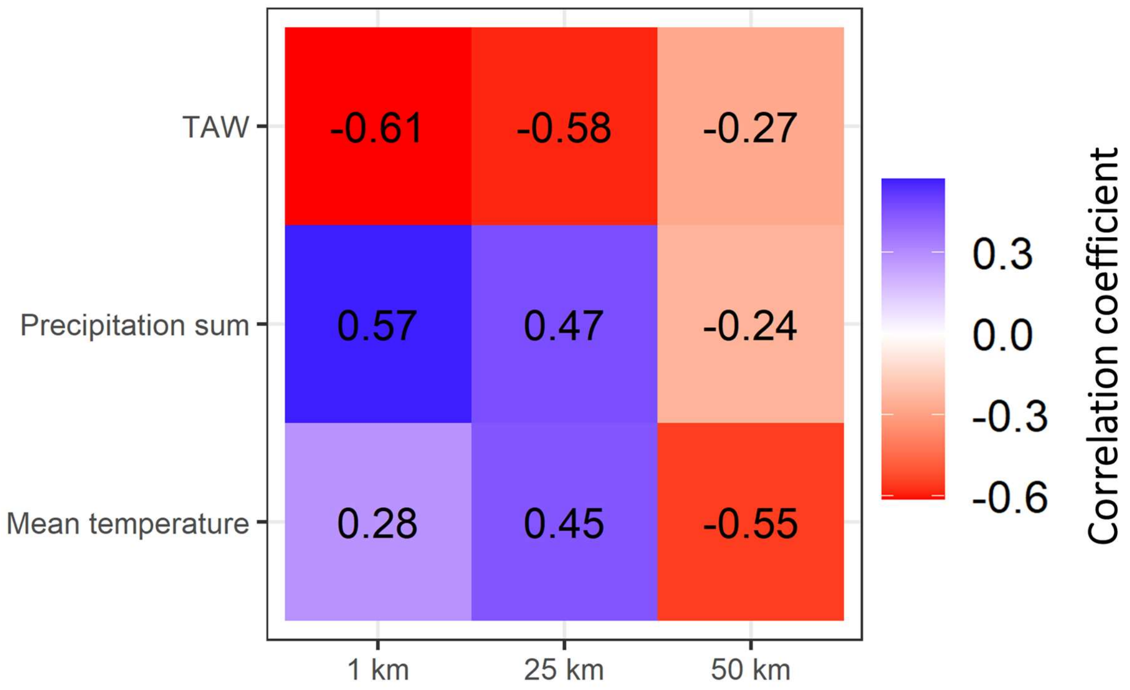

Supplementary figure 2. Correlation coefficient between the difference in simulated yield among field and other resolution-sources and the difference in inputs (mean temperature, precipitation sum, soil total available water [TAW]) between field and other resolution-sources for the period 2001-2010 (during growing season).

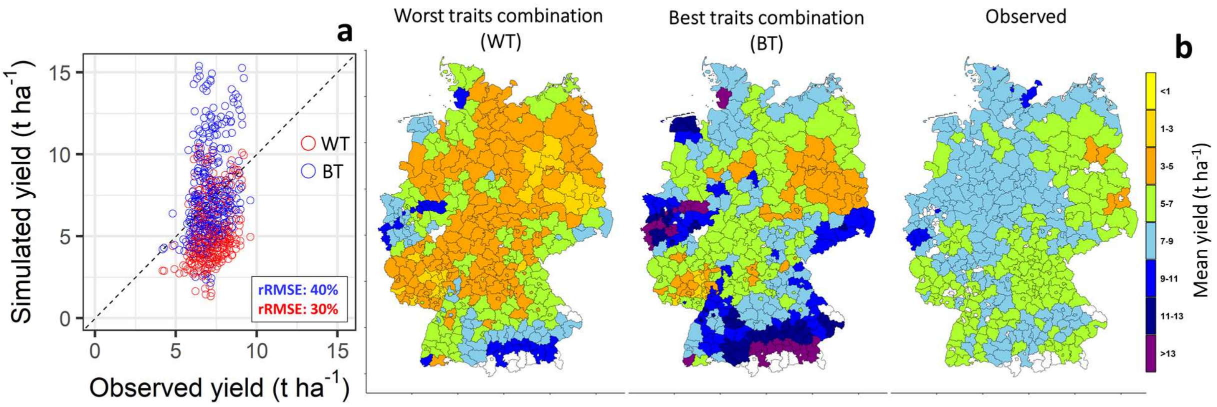

Supplementary figure 3. 1:1 plot (a), and the spatial pattern (b) of simulated (using the best and worst trait combinations) versus observed wheat yield at the NUTS3 scale for the period 2001-2010 across Germany. Simulated yield aggregated to NUTS3 scale from simulations at 1 km resolution. Simulated yield aggregated to NUTS3 scale from simulations at 1 km resolution. rRMSE: relative root mean squared error.

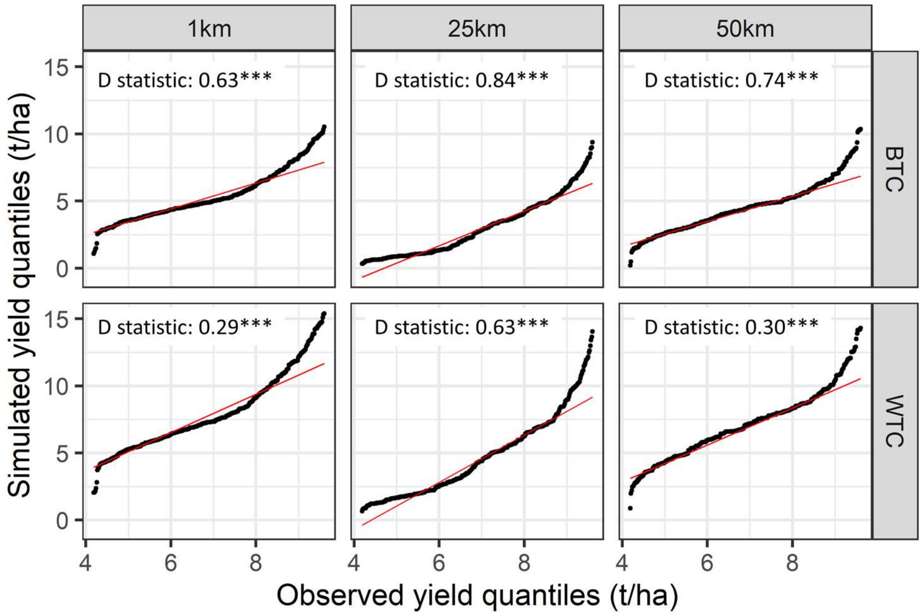

Supplementary figure 4. Q-Q plots comparing mean (2001-2010) simulated wheat yields (across different spatial resolutions [1 km, 25 km, 50 km]) to mean observed yields and trait combinations (BTC: Best Trait Combination, WTC: Worst Trait Combination). The red diagonal line represents perfect agreement between simulated and observed distributions. D statistics from Kolmogorov-Smirnov tests are shown for each plot, with *** indicating $p < 0.001$. Deviations from the diagonal line suggest differences between simulated and observed yield distributions, with larger D statistics indicating larger discrepancies.

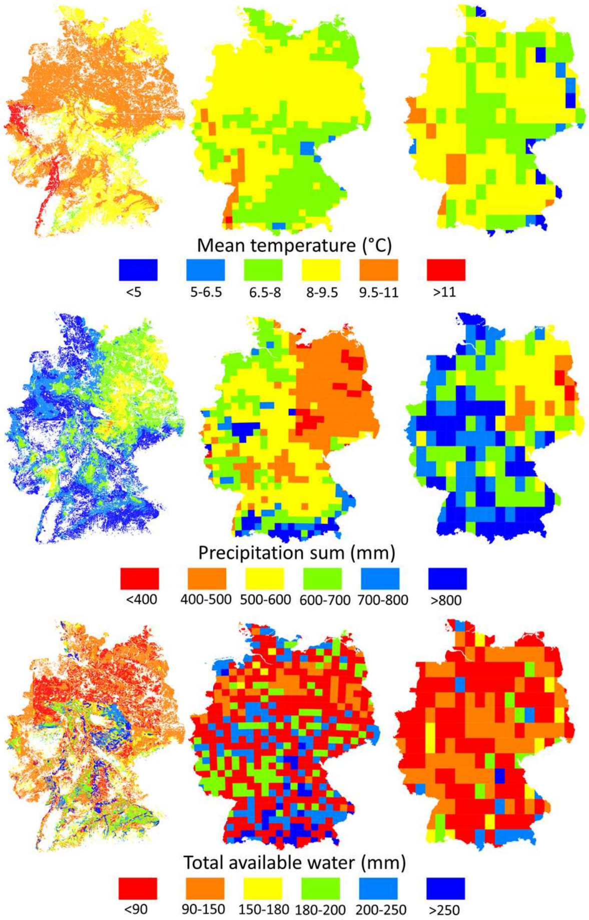

Supplementary figure 5. Spatial patterns of mean temperature, precipitation sum, and total available water during the period 2001-2010 across Germany.

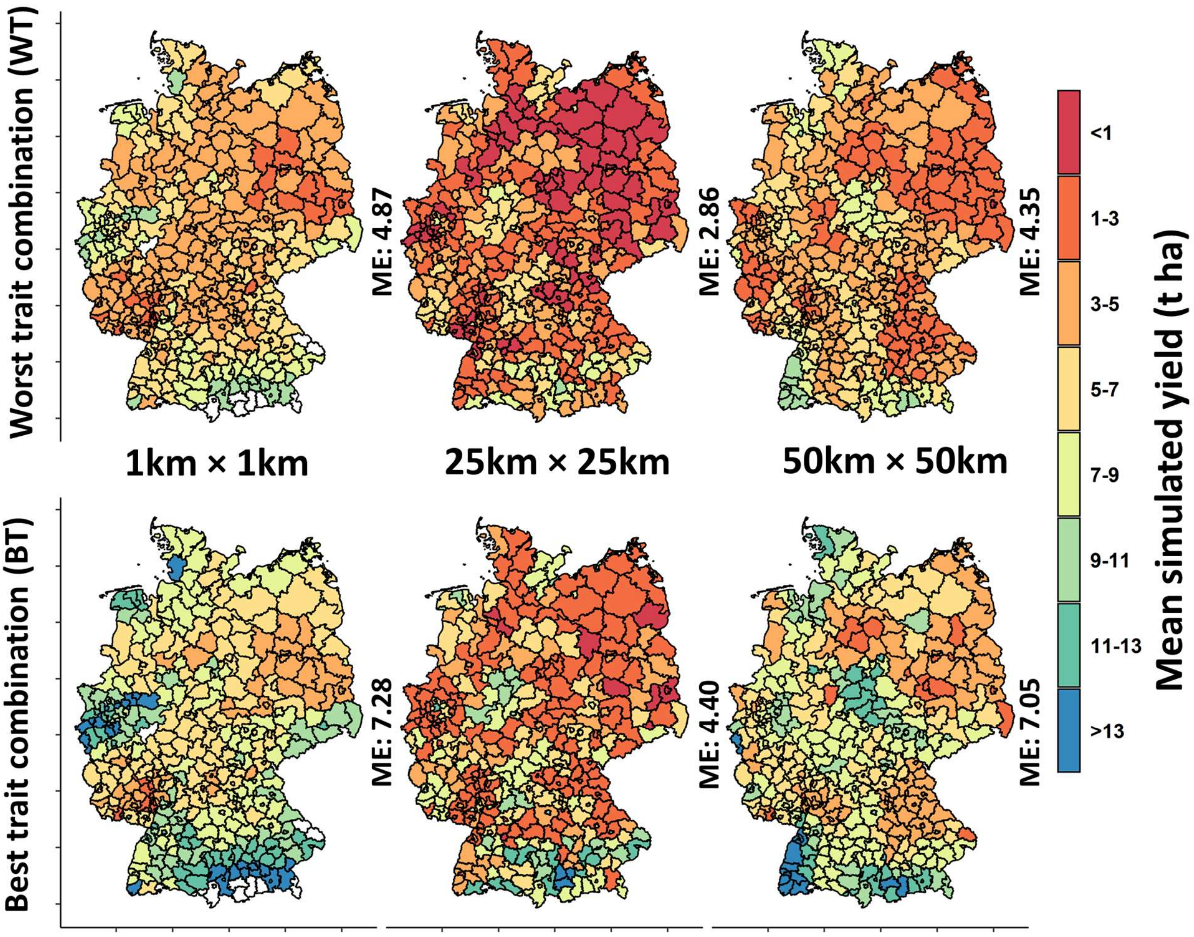

Supplementary figure 6. The spatial pattern of mean simulated yield aggregated from 1 km, 25 km, and 50 km to the NUTS3 scale in the period 2001-2010. ME: mean.
